# Statistics for the analysis of molecular dynamics simulations: providing *P* values for agonist-dependent GPCR activation

**DOI:** 10.1038/s41598-020-77072-4

**Published:** 2020-11-17

**Authors:** Agustín Bruzzese, James A. R. Dalton, Jesús Giraldo

**Affiliations:** 1 Laboratory of Molecular Neuropharmacology and Bioinformatics, Unitat de Bioestadística and Institut de Neurociències, Universitat Autònoma de Barcelona, 08193 Bellaterra, Spain; 2 Unitat de Neurociència Traslacional, Parc Taulí Hospital Universitari, Institut d’Investigació i Innovació Parc Taulí (I3PT), Institut de Neurociències, Universitat Autònoma de Barcelona, Bellaterra, Spain; 3 Centro de Investigación Biomédica en Red de Salud Mental, CIBERSAM, Instituto de Salud Carlos III, Madrid, Spain

**Keywords:** Biophysics, Computational biology and bioinformatics, Drug discovery, Structural biology

## Abstract

Molecular dynamics (MD) is the common computational technique for assessing efficacy of GPCR-bound ligands. Agonist efficacy measures the capability of the ligand-bound receptor of reaching the active state in comparison with the free receptor. In this respect, agonists, neutral antagonists and inverse agonists can be considered. A collection of MD simulations of both the ligand-bound and the free receptor are needed to provide reliable conclusions. Variability in the trajectories needs quantification and proper statistical tools for meaningful and non-subjective conclusions. Multiple-factor (time, ligand, lipid) ANOVA with repeated measurements on the time factor is proposed as a suitable statistical method for the analysis of agonist-dependent GPCR activation MD simulations. Inclusion of time factor in the ANOVA model is consistent with the time-dependent nature of MD. Ligand and lipid factors measure agonist and lipid influence on receptor activation. Previously reported MD simulations of adenosine A2a receptor (A2aR) are reanalyzed with this statistical method. TM6–TM3 and TM7–TM3 distances are selected as dependent variables in the ANOVA model. The ligand factor includes the presence or absence of adenosine whereas the lipid factor considers DOPC or DOPG lipids. Statistical analysis of MD simulations shows the efficacy of adenosine and the effect of the membrane lipid composition. Subsequent application of the statistical methodology to NECA A2aR agonist, with resulting *P* values in consistency with its pharmacological profile, suggests that the method is useful for ligand comparison and potentially for dynamic structure-based virtual screening.

## Introduction

Molecular dynamics (MD) simulations is an established computational tool for the examination of the conformational flexibility of molecules, in particular proteins^1^. G protein-coupled receptors (GPCRs) are membrane proteins responsible for signal transduction from outside to inside the cell. Thus, the healthy or pathologic state of living organisms greatly depends on the correct or anomalous functional molecular state of GPCRs. This is translated into the fact that GPCRs are the target for about one third of current marketed medicines^2^.

GPCRs, also known as 7-transmembrane (7-TM) receptors, have in common that they all bear seven transmembrane helices which are connected by three extra- and three intracellular loops. A number of biophysical approaches amongst them crystallography and different spectroscopic techniques have studied the conformational changes associated to the activation of GPCRs^3^. Particularly in Class A GPCRs, receptor activation involves a large outward movement of TM helix 6 (TM6) from the central TM3 and a smaller inward movement of TM7 as relevant mechanistic conformational features^4^.

To reveal GPCR activation conformational features, MD simulations need at least µs-length trajectories and the use of several replicas to provide sufficient confidence to computational results. It is worth noting that, although starting from the same conformational arrangement and applying identical experimental conditions, two independent trajectories can evolve differently leading to dissimilar results. Also importantly is that MD simulations are inherently time-dependent and, consequently, the time factor should be present in their statistical analysis.

Despite the wide use of MD simulations there is not a unified approach for their analyses. Different multivariate statistical analyses such as cluster and principal component analysis and, more recently, machine learning approaches are being applied^5,6^. Here we present a multiple-factor analysis of variance (ANOVA) with repeated measurements on the time factor, a classical statistical approach which makes use of the time-dependent nature of MD simulations and quantifies the statistical effect that several experimental conditions may have on the activation capability of a receptor. The main advantages of the approach are (1) its general practicality, as it is included in most statistical packages, and (2) the computational production of *P* values, which removes subjectivity from conclusions.

## Results and discussion

**Figure 1:**
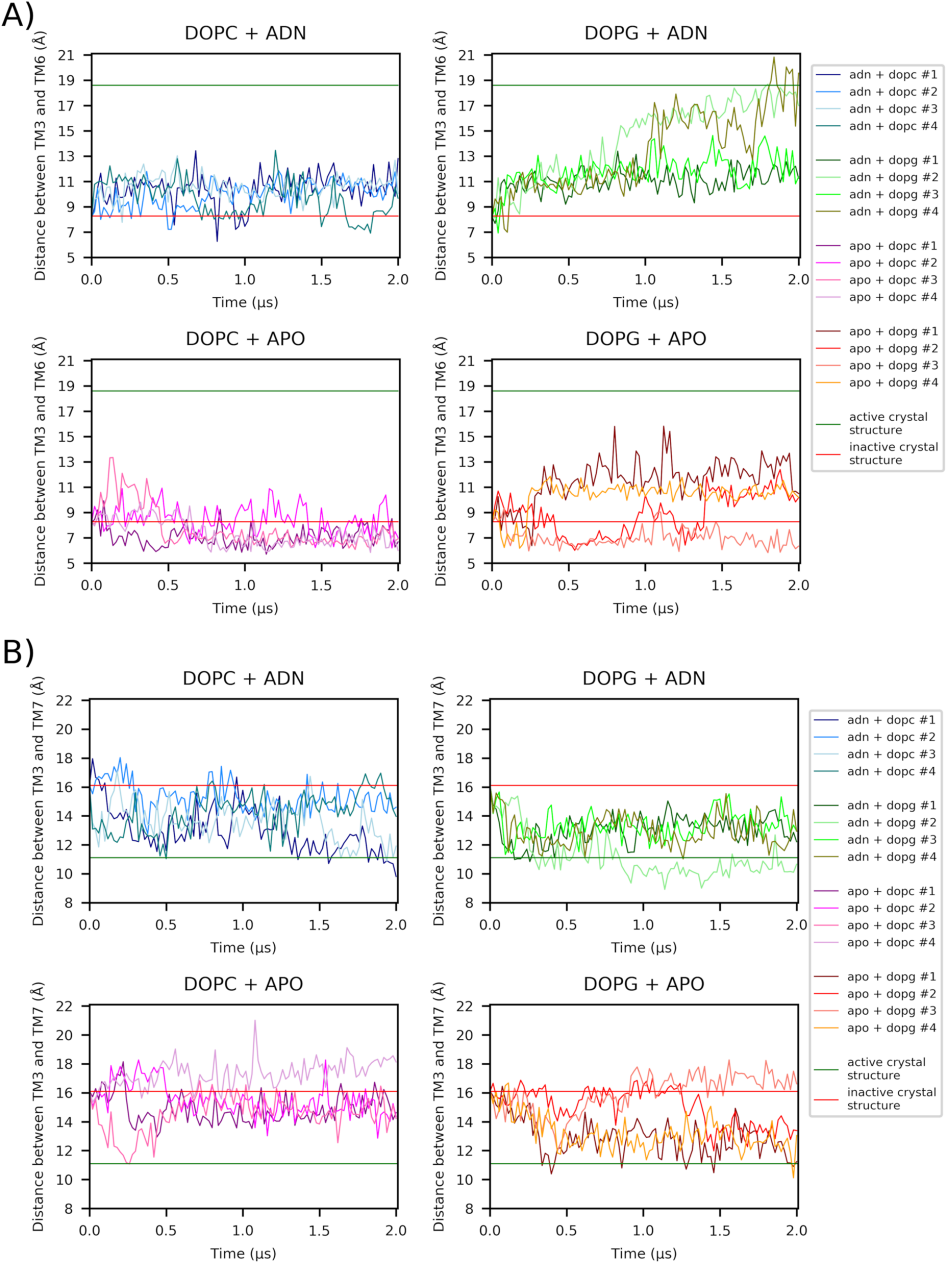
Examination of structural features depicting adenosine-dependent receptor activation in A2aR from 2 µs -length MD simulations. Lipid (DOPC, DOPG) and ligand (adenosine, APO) experimental conditions were considered. 4 replicas for each condition combination were run. Structures were taken every 0.02 µs. (**A**) The TM3–TM6 distance is measured between Cα atoms of R102^3.50^ and E288^6.30^. (**B**) The TM3–TM7 distance is measured between Cα atoms of R102^3.50^ and Y288^7.53^. Horizontal TM3–TM6 and TM3–TM7 red lines correspond to the distances between the aforementioned respective atoms for the inactive receptor (PDB entry: 4EIY)^16^ whereas horizontal green lines correspond to the distances for the active receptor (PDB entry: 6GDG)^17^. ADN stands for adenosine. Figures adapted from^7^.

In the present article we reanalyze a recent study of ours in which the activation of the adenosine A2a Class A GPCR (A2aR) was examined^7^. In this study, 2 µs-length MD simulations of A2aR under 2 experimental conditions: absence/presence of endogenous adenosine and DOPC (1,2-dioleoyl-sn-glycerol-3-phosphocholine)/DOPG (1,2-dioleoyl-sn-glycerol-3-phosphoglycerol) lipid environment were performed^7^. To provide sufficient variability, 4 replicas were run in each condition value. Figure 1 shows the trajectories of each of the replicas in each of the experimental conditions for the two main variables reflecting the receptor activation process, namely, the distances between TM3 and TM6 (TM3–TM6) and between TM3 and TM7 (TM3–TM7). Values were collected every 0.02 µs, totalizing 100 time-points for each replica. Visual inspection of Fig. 1A shows the effect of both the ligand (agonist, in this case) and lipid factors on TM3–TM6: adenosine increases the magnitude of this variable over the apo receptor and, similarly, does DOPG with respect to DOPC. The same overall effects, though in an opposite direction and lower degree, happen for TM3–TM7 (Fig. 1B): adenosine decreases the TM3–TM7 distance over the apo receptor and, seemingly, does DOPG with respect to DOPC. However, there are differences between the 4 replicas within each of the (lipid, ligand) experimental conditions. We may all agree that the visual variability observed between and within curves in Fig. 1 needs quantification and proper statistical analysis to provide meaningful conclusions.

**Figure 2:**
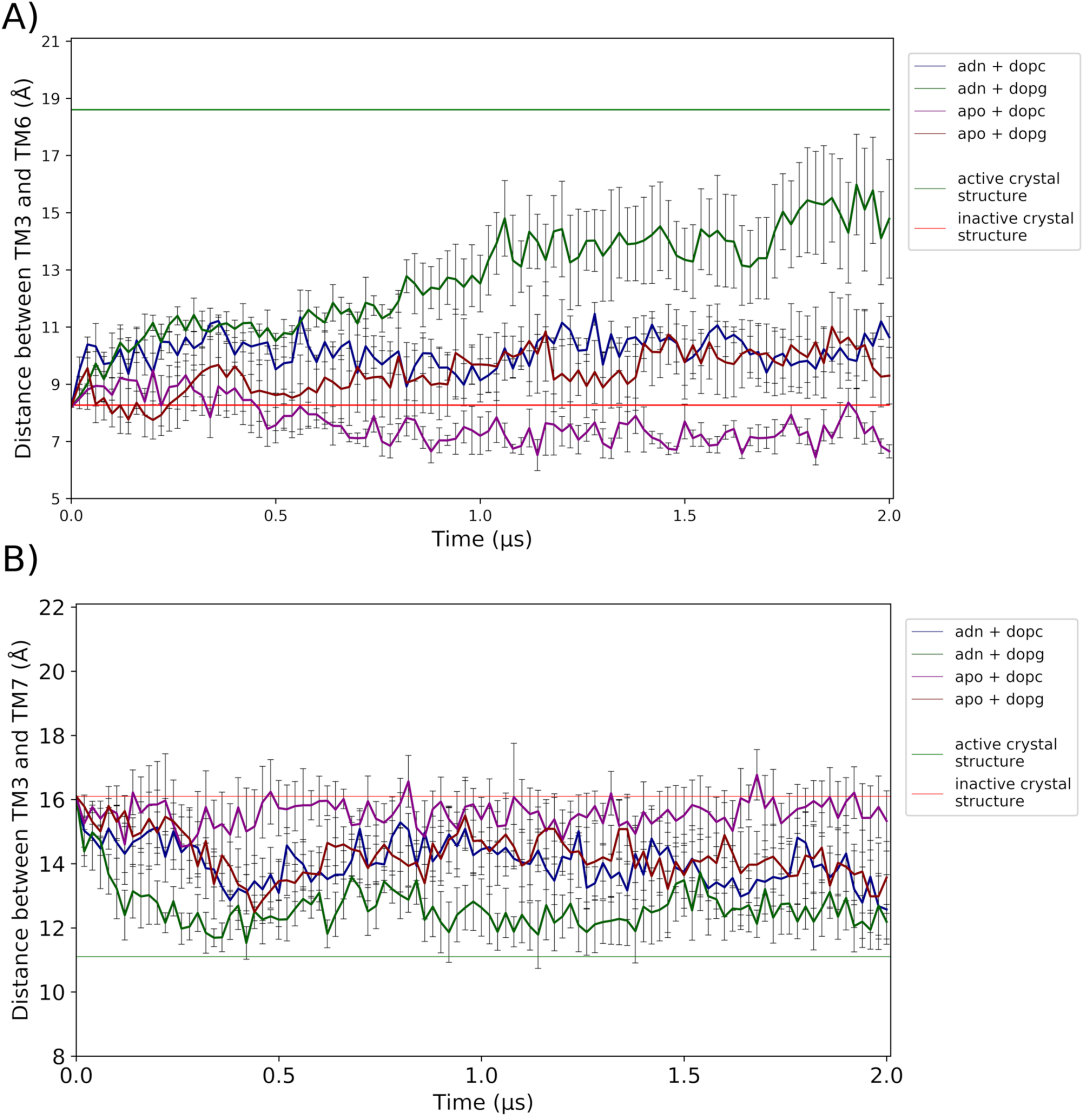
Descriptive statistics, mean and standard error of the mean (SEM), for the sampled 4 replicas in each of the conditions (lipid, adenosine) depicted in Fig. 1. (**A**) TM3–TM6 distance. (**B**) TM3–TM7 distance. Horizontal lines have the same definition as in in Fig. 1. ADN stands for adenosine.

Figure 2 depicts means and standard errors of the mean (SEM) along time for the 4 replicas included in each of the (ligand, lipid) experimental conditions of the MD simulations for TM3–TM6 and TM3–TM7 variables. To statistically assess the variability between and within replicas a three-factor (time, ligand, lipid) ANOVA with repeated measurements on the time factor was performed. Table 1 shows the *P* values for the tested effects on the selected variables, where an MD simulation trajectory or replica takes the sense of subject or experimental unit in the common statistical language.

**Table 1:** Three-factor (time, ligand, lipid)-ANOVA of receptor activation with repeated measurements on time factor.

	TM3–TM6	TM3–TM7
**Between-trajectory effects**		
Lipid	*P* = 6.18 × 10^−3^	*P* = 0.0411
Ligand	*P* = 7.98 × 10^−4^	*P* = 0.0201
Lipid × ligand	*P* = 0.5669	*P* = 0.9288
**Within-trajectory effects**		
Time	*P* = 6.10 × 10^−10^	*P* = 2.11 × 10^−3^
Time × lipid	*P* = 5.34 × 10^−34^	*P* = 0.9912
Time × ligand	*P* = 1.47 × 10^−7^	*P* = 0.9984
Time × lipid × ligand	*P* = 0.9473	*P* = 0.2869

Ligand: adenosine (present/absent). Lipid: DOPC/DOPG. Statistical analysis of data depicted on Figs. 1 and 2.

On the analysis of TM3–TM6, Table 1 shows that both Lipid and Ligand variables have significant effects (*P* = 0.0062 and *P* = 0.0008, respectively). On the contrary, the Lipid × Ligand interaction is not significant (*P* = 0.5669), meaning that the effect of the ligand is similar in both lipid environments. When analyzing the within-trajectory effects, we see that Time is significant (*P* < 0.0001) and also both Time × Lipid (*P* < 0.0001) and Time × Ligand (*P* < 0.0001) interactions, meaning that there is an increase in average of TM3–TM6 with time and that this increase depends on the lipid and the ligand compositions. Finally, the absence of statistical significance of the Lipid × Ligand interaction maintains this value when time is also included in the interaction (*P* = 0.9473). Translating probability values into pharmacological concepts, we can say that DOPG environment facilitates receptor activation more than DOPC and that adenosine is more efficacious than the free receptor in inducing an active conformation, irrespective of the membrane lipid composition. The activation of the receptor occurs progressively along time and the effect along time is not the same for each of the lipids and also for adenosine in comparison to the free receptor. Finally, the effect of the lipid along time happens for both adenosine-bound and the free receptor.

On the analysis of TM3–TM7, we see that, similarly to the TM3–TM6 evaluation, both Lipid and Ligand variables have significant effects (*P* = 0.0411 and *P* = 0.0201, respectively). Analogously also to TM3–TM6, the Lipid × Ligand interaction is not significant (*P* = 0.9288). It is worth noting that the *P* values for the Lipid and Ligand effects are lower in TM3–TM7 than in TM3–TM6, which is in agreement with depicted curve profiles and the general consensus that TM6 outward movement is the principal structural feature characterizing Class A GPCR activation. When analyzing the within-trajectory effects, we see that Time is significant (*P* = 0.0021) but, contrary to TM3–TM6, neither Time × Lipid (*P* = 0.9912) nor Time × Ligand (*P* = 0.9984) interactions are significant. Or in other words, neither the effect of lipid nor the effect of ligand significantly increase with time. Finally, the absence of statistical significance of the Lipid × Ligand interaction maintains this value when time is also included in the interaction (*P* = 0.2869). We may attribute the differences in statistical significance between TM3–TM6 and TM3–TM7 variables in the Time interaction effects to the observed large effects on the TM3–TM6 variable of the inclusion of both Ligand = adenosine and Lipid = DOPG conditions (compare Fig. 2A,B).

Interestingly, the Ligand factor provides a statistical comparison between the free receptor and an agonist-bound receptor; in the present case, adenosine. Thus, a correspondence can be made between the *P* value and the concept of intrinsic efficacy^8^; in the present case, for two different structural features: TM3–TM6 and TM3–TM7, with the former as the most indicative of receptor activation.

**Figure 3:**
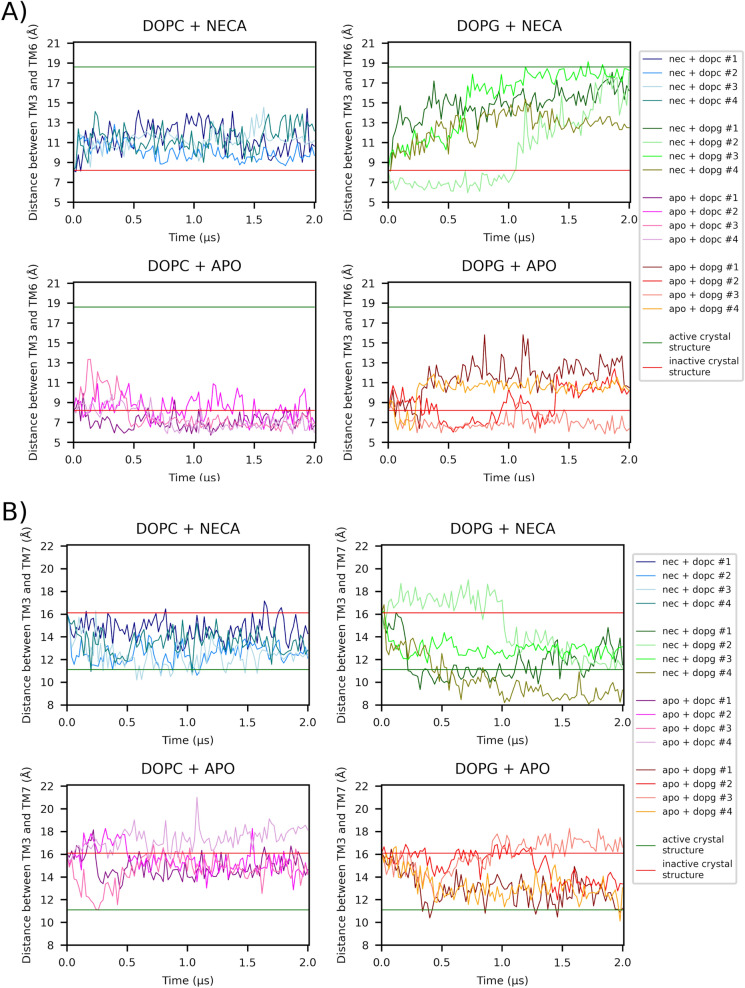
Examination of structural features depicting NECA-dependent receptor activation in A2aR from 2 µs -length MD simulations. Lipid (DOPC, DOPG) and ligand (NECA, APO) experimental conditions were considered. 4 replicas for each condition combination were run. Structures were taken every 0.02 µs. (**A**) The TM3–TM6 distance is measured between Cα atoms of R102^3.50^ and E288^6.30^. (**B**) The TM3–TM7 distance is measured between Cα atoms of R102^3.50^ and Y288^7.53^. Horizontal TM3–TM6 and TM3–TM7 red lines correspond to the distances between the aforementioned respective atoms for the inactive receptor (PDB entry: 4EIY)^16^ whereas horizontal green lines correspond to the distances for the active receptor (PDB entry: 6GDG)^17^. Figures adapted from Ref ^7^.

**Figure 4:**
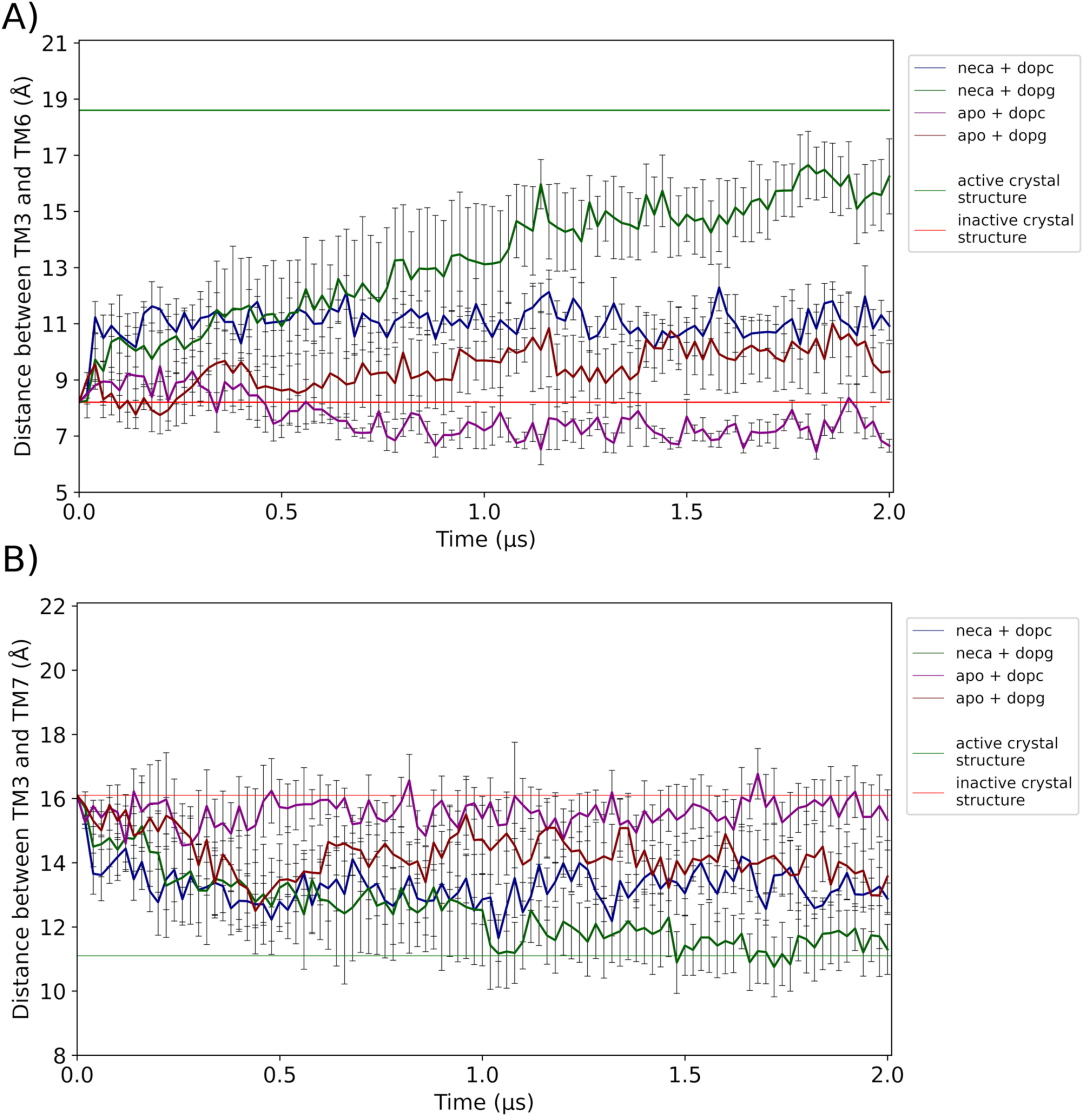
Descriptive statistics, mean and standard error of the mean (SEM), for the sampled 4 replicas in each of the conditions (lipid, NECA) depicted in Fig. 3. (**A**) TM3–TM6 distance. (**B**) TM3–TM7 distance. Horizontal lines have the same definition as in in Fig. 1 and 3.

To investigate whether the proposed statistical methodology can be useful for drug comparison, we selected a set of trajectories of NECA (5′-*N*-ethylcarboxamidoadenosine) A2aR agonist which were run under the same conditions of trajectory-length, lipid composition and number of replicas as those of adenosine (Figs. 3 and 4). As in the case of adenosine, these trajectories are described individually in Ref.^7^. Considering that NECA is a more potent agonist than adenosine one would expect that the performed statistics would reflect this pharmacological feature. Table 2 displays the *P* values of NECA MD simulations, which can be compared with those of adenosine in Table 1. If we focus on those factors related with ligand, we see that, in the case of TM3–TM6 dependent variable, the *P* value for the Ligand factor is slightly lower for NECA (*P* = 7.61 × 10^−4^) in comparison with adenosine (*P* = 7.98 × 10^−4^). More important is the Time × Ligand effect: *P* = 3.97 × 10^−14^ for NECA and *P* = 1.47 × 10^−7^ for adenosine. Moreover, in the case of the TM3–TM7 dependent variable, the *P* values for the Ligand factor are *P* = 0.0174 and *P* = 0.0201 for NECA and adenosine, respectively, whereas for the Time × Ligand effect the *P* values are *P* = 0.5272 and *P* = 0.9984 for NECA and adenosine, respectively. Overall, we see that the effect of ligand factor is stronger for NECA than for adenosine in agreement with their agonist potency profiles.

**Table 2:** Three-factor (time, ligand, lipid)-ANOVA of receptor activation with repeated measurements on time factor.

	TM3–TM6	TM3–TM7
**Between-trajectory effects**		
Lipid	*P* = 0.0296	*P* = 0.1860
Ligand	*P* = 7.61 × 10^−4^	*P* = 0.0174
Lipid × ligand	*P* = 0.7809	*P* = 0.7299
**Within-trajectory effects**		
Time	*P* = 1.84 × 10^−14^	*P* = 3.55 × 10^−6^
Time × lipid	*P* = 5.15 × 10^−45^	*P* = 0.0045
Time × ligand	*P* = 3.97 × 10^−14^	*P* = 0.5272
Time × lipid × ligand	*P* = 0.8651	*P* = 0.5814

Ligand: NECA (present/absent). Lipid: DOPC/DOPG. Statistical analysis of data depicted on Figs. 3 and 4.

As a hypothesis to be tested with further studies, it could be proposed that, by generalizing the previous results, the herein shown formalism could be applied in high throughput MD simulations of large collections of ligands. In doing so, the *P* values of the MD simulations could be later used as a statistical descriptor to rank agonists according to their capability to activate the receptor (at in silico level and with the inherent limitations of a computational study). Considering that each agonist is compared with the free receptor, this is a way to approach the agonist efficacy concept, from MD simulations and within a statistical perspective. Ranked *P* values of a set of agonists for a particular receptor in combination with the structure of these ligands could be used as a component of following quantitative structure–activity studies. These studies could lead to new drug design. In this respect, MD simulations could provide a time-dependent framework for virtual screening purposes. This virtual screening can be made either directly through MD simulations of the chosen ligands or indirectly by ligand docking on selected receptor structures from MD simulations of apo receptors or ligand-bound receptors—the latter in the case of evaluating allosteric modulators. In this way, MD-based virtual screening can be a complement to the more classical structure-based virtual screening, which targets static crystal structures^9–11^.

MD is a computational technique especially appropriate for addressing the flexibility of the target proteins, GPCRs in the present study. It seems logical that, in general, considering the flexibility of the target can increase the probability of identifying new ligands. However, whether this is really an advantage for GPCR drug discovery is a current debate. In a recent publication on the performance of virtual screening against GPCR homology models^12^, in which binding site plasticity was considered by including ensembles of structures, it was shown that MD refinement resulted in moderate improvements of structural accuracy and the virtual screening performance of snapshots was either comparable to or worse than that of the raw homology models^12^. However, and from a different perspective, the methodological approach herein shown is focused on the statistical significance of structural features reflecting receptor activation. To this end, we are proposing multiple-way ANOVA for the statistical analysis of those MD simulations that are addressed to allow the distinction between agonists, neutral antagonists and inverse agonists (through statistical comparison with the MD simulations of the apo receptor). Molecular docking screening focused on specific ligand sets has been considered elsewhere^13^. In this study, a large library virtual screen against an active β_2_-adrenergic receptor (β2AR) crystal structure returned agonists exclusively and with a high hit rate^13^. However, it seems that, in general, structural information from active receptors is not transferrable to other receptors despite reasonable sequence identity. When the same authors used the β2AR active state as a template for the construction of a dopamine D2 receptor (DRD2) activated model, although both receptors share 42% sequence identity, virtual screening was not satisfactory: few weak agonists mixed with an inverse agonist were selected from the modeled DRD2 active state^13^. Thus, it seems plausible to hypothesize that MD simulations can potentially be an appropriate complement for virtual screening based on static structures and, interestingly, they can be especially addressed to distinguish between agonists, neutral antagonists and inverse agonists, particularly, in those cases in which the crystal active receptor state has not been determined. We must admit that the computational cost of the required MD simulations can be a limiting barrier of the methodology if is intended for high throughput screening. However, it may be accepted that, with a sustained exponential growth in computational power, long and massive MD simulations can be accessible in the near future.

## Concluding remarks

To summarize, we conclude that multiple-factor ANOVA with repeated measurements on the time factor can be a useful statistical technique for the analysis of MD simulations of ligand-bound GPCRs under various experimental conditions. The proposed methodology has the benefit of including *P* values for assessing statistical significance of testing hypotheses, in particular agonist efficacy. In addition, resulting *P* values can provide a probabilistic framework for dynamic structure-based virtual screening if the limitation of the computational cost is overcome. The methodology can be extended to systems other than GPCRs by defining the appropriate dependent variables.

## Methods

Three-factor (time, ligand, lipid) analysis of variance (ANOVA) is proposed for the statistical analysis of GPCR MD simulations to assess receptor activation. This method was selected because it is a confirmatory statistical technique in which a hypothesis concerning receptor activation can be tested and associated *P* values obtained. Either the TM6–TM3 or the TM7–TM3 distances were chosen as dependent variables because it is known that their respective increase or decrease with respect to the inactive receptor state are indicative of receptor activation. To allow for variability, 4 trajectories or replicas for each of the selected ligand and lipid factors were run. Each trajectory is considered as a subject or experimental unit in the statistical analysis performed. Special attention was paid to the fact that an MD simulation inherently involves time-dependent data. Thus, we have time-dependent values for TM6–TM3 and TM7–TM3 along the trajectories. The length of each of the trajectories was 2 µs. Values of each of the dependent variables were collected every 0.02 µs, totalizing 100 time-points for each replica. Thus, we have 100 values for each trajectory providing information on how TM6–TM3 and TM7–TM3 evolve along time for each combination of ligand and lipid values (see below). Time was included in the ANOVA model as a repeated-measurement factor as each trajectory is repeatedly measured along time. To evaluate the efficacy of adenosine, the Ligand factor with 2 values (adenosine present or absent) was included. Note that when analyzing the trajectories corresponding to NECA agonist the values of the Ligand factor are NECA present or absent. The lipid composition of the membrane (Lipid) was included in the ANOVA model as the third factor. Two lipid values were considered: DOPC or DOPG. This would allow to test the lipid effect on receptor activation and whether the agonist capability depends or not on the lipid environment. 24 trajectories (3 (ligand) × 2 (lipid) × 4 (replica)) were included in the analyses. The TM3–TM6 distance was measured between Cα atoms of R102^3.50^ and E288^6.30^. The TM3–TM7 distance was measured between Cα atoms of R102^3.50^ and Y288^7.53^ (see Ref^14^ for numbering notation). In order for ANOVA to be applied some assumptions need to be satisfied: (1) the distribution of values should be normally distributed and (2) the groups should come from populations with equal variances (homogeneity of variances). The normality assumption for each group of 4 replicas was verified through the Shapiro–Wilk test for normality whereas the homogeneity of variances was tested by the F-distribution. We checked the applicability conditions at each time-point of the trajectories corresponding to adenosine system. For the TM3–TM6 dependent variable, the normality assumption was accomplished in 93% of the time points whereas the homogeneity of variances assumption was satisfied in 88% of time points. For the TM3–TM7 dependent variable, the normality assumption was accomplished in 94% of the time points whereas the homogeneity of variances assumption was satisfied in 96% of time points. Considering the broad compliance of the assumptions and the robustness of the ANOVA method, which allows its application even if the assumptions are not fully accomplished^15^, we think the herein proposal of ANOVA formalism for MD simulations analysis is justified. SAS 9.4 statistical package (SAS Institute Inc., Cary, NC, USA) was used for statistical analyses. *P* values lower than 0.05 were considered statistically significant.
